# Progressing the understanding of chronic illness and its treatment: A post-human, ethological understanding of haemodialysis

**DOI:** 10.1177/13634593231200126

**Published:** 2023-09-14

**Authors:** Victoria Cluley, Helen Eborall, Katherine Hull, Niamh Quann, James O Burton

**Affiliations:** University of Nottingham, UK; University of Edinburgh, UK; University of Leicester, UK; University Hospitals of Leicester NHS Trust, UK; University of Leicester, UK; University of Leicester, UK; University Hospitals of Leicester NHS Trust, UK; Loughborough University, UK

**Keywords:** assemblage, end-stage kidney disease, haemodialysis, new materialism, posthumanism

## Abstract

Haemodialysis is a common treatment option offered internationally for people requiring kidney replacement therapy. Research exploring haemodialysis is predominantly clinical and quantitative, and improvements to its provision and receipt tends also to be clinically focused. In recent years, however, a number of studies have sought to explore the lived experience of haemodialysis. These studies tend to use semi-structured interviews and present descriptive findings. Such findings serve to raise the profile of patient perspectives and encourage thinking beyond the clinical gaze. To progress this, we apply a post-humanism approach to the understanding of the receipt of haemodialysis. Drawing on findings from a study to explore the experience and impact of in-centre, daytime, haemodialysis we follow Fox and Alldred’s ethological toolkit to provide a post-human analysis of haemodialysis. In doing so we argue that haemodialysis exists as a heterogenous and changeable assemblage of multiple and fluid, human and non-human factors that has the capacity to affect. Here we outline this post-human approach and the impact it has for understanding not just haemodialysis but also the receipt of treatment for other chronic illnesses.

## Introduction

In this paper we apply an ethological approach to contribute a holistic understanding of the impact of haemodialysis. We propose that haemodialysis functions as an assemblage of fluid and heterogenous, human and nonhuman factors that has the capacity to be affective and affected. Understanding this heterogeneous assemblage of human and nonhuman factors and how it both produces and affects the haemodialysis experience is essential to the improvement of care offered to those in receipt of haemodialysis. Understanding treatment options beyond what is clinically known about them through the experience of those who receive them is fundamental to the improvement of health and social care provision, treatment options and individual lived experience (Kierans, 2005; Morden et al., 2017). As it stands, qualitative research that seeks to develop the understanding of chronic illness and its treatment tends to be conducted from a social constructionist or embodied/phenomenological starting point (Cluley et al., 2023a). Such research has successfully expanded the understanding of chronic illness by questioning the dominance of a medical model of health and introducing lived/embodied experience, social environmental and cultural factors. Here we seek to progress this work by applying a post-human lens to the understanding of chronic illness and its treatment. We present and reflect on the use of Fox and Alldred’s ethological toolkit to conduct a secondary analysis of findings taken from qualitative work conducted as part of the NightLife study (ISRCTN87042063).

It is hoped that in unpacking the process and experience of haemodialysis in this way and showing the affects of this, will encourage the use of an ethological approach to understanding other chronic illnesses and treatment options. To do this, we first provide some background regarding end-stage kidney disease, the haemodialysis process and previous literature addressing the experience of this chronic illness and treatment option. The theoretical approach is then outlined, and the post-human method of ethology is introduced alongside the methodological approach taken in the NightLife study. We present and discuss the research findings our secondary analysis in line with the ethological approach.

### End-stage kidney disease and haemodialysis

End-stage kidney disease means that the kidneys no longer function sufficiently to remove toxins from the blood to maintain life and the kidney function is not recoverable. Kidney replacement therapy aims to sustain life through either transplantation or dialysis (the process of artificially removing excess water, toxins and solutes). Dialysis is a long-term and intensive treatment, requiring daily or alternate day scheduling, taking place either at home or in a healthcare setting. Although it varies between countries and healthcare systems, the burden of kidney failure is significant and growing. Globally, more than 800 million people live with kidney disease and of those, nearly 4 million have kidney failure that requires some kind of kidney replacement therapy (KRT). Haemodialysis is the most common form of KRT, representing 69% of all KRT and 89% of all dialysis (Bello et al., 2022).

The haemodialysis process is hinged on a specific infrastructure to ensure safe and effective treatment, including: electricity supply, ultrapure water treatment and waste disposal. Haemodialysis is a complex treatment; blood is passed through an artificial kidney, known as the dialyser, to remove water, solutes and toxins. For effective dialysis, the blood flow rate needs to be high over a significant period of time. Typically, an individual on haemodialysis will have around 400–500 mL of blood removed and returned to their body every minute over 3.5–5 hours. The standard prescription of in-centre haemodialysis is thrice weekly and adherence is required for the rest of the individual’s life or until successful transplantation. However, many individuals are not eligible for kidney transplantation due to a lack of access to organs as well as other patient, health-care and system related barriers (see Venkataraman and Kendrick, 2020 for a more detailed explanation).

To enable the removal and return of blood, sufficient vascular access is required; ability to achieve high blood flow rates and accessible for connection to the dialysis machine. This is typically achieved through an arteriovenous fistula, whereby an artery and a vein are connected together to create a robust vessel that will tolerate regular insertion of needles (one for the removal and one for the return of blood). Arteriovenous fistulae are usually located in the arm. If the requirement for haemodialysis has occurred before arteriovenous fistulae formation, or this is not an option, haemodialysis vascular access will occur through a tunnelled catheter, also known as a ‘line’ or ‘permcath’. The tunnelled catheter is located in either the neck or groyne, after being placed in a major large vein (i.e. internal jugular vein in the neck, femoral vein in the groyne). Occasionally, a patient may have an arteriovenous graft. In this case, the patient does not have suitable blood vessels for an arteriovenous fistula, and so an artificial watertight tube is used to connect an artery and a vein.

Haemodialysis can take place in a hospital setting or at home, however, the majority of people receiving haemodialysis in the UK receive in-centre dialysis. In the UK for example, around 24,000 people were receiving incentre haemodialysis by the end of 2020 compared with 1377 who received dialysis at home (UK Renal Registry (UKRR), 2022). Owing to the temporal, physical and psychological demands of haemodialysis, this treatment option has a significant impact on the lives of those receiving it.

### Current literature

As it stands qualitative research addressing the receipt of haemodialysis is limited in comparison to the quantitative evidence. Studies tend to focus on quality of life and health outcomes (McKeaveney et al., 2023; Young et al., 2000). Quantitative studies measuring quality of life have found end-stage kidney disease and its treatments negatively affect quality of life, with patients reporting reduced vitality, physical function and mental health (Chuasuwan et al., 2020; Cleary and Drennan, 2005). Qualitative studies have highlighted the frustrations and challenges experienced by dialysis patients, such as changes to employment status, financial challenges, changes to living arrangements, the impact on relationships, stress, anxiety and low mood, and reduced social activity (Cluley et al., 2023a; DePasquale et al., 2020; Kierans and Maynooth, 2001; Park et al., 2015; Roberti et al., 2018). These studies have, in the main, used a combination of semi-structured interviews and thematic analysis, and present descriptive findings with limited theoretical explication. While descriptive, qualitative studies are useful in raising the profile of patient experience, it is also important to explore these findings theoretically in order to progress the contribution that can be made to the understanding of the lived experience of haemodialysis and thereby work towards a holistic approach to the improvement of treatment and care.

Qualitative studies addressing end-stage kidney disease and/or haemodialysis tend to be conducted from a social constructionist perspective, whereby social and cultural norms are afforded importance in the construction of events and experiences. While this position is rarely, if ever, explicitly stated, this epistemological position is generally implicit within the research design chosen, which as outlined above, more often than not involves semi-structured interviews coupled with a form of thematic analysis.

Where social theory has been used to make sense of the experience of haemodialysis, this has tended to be from a phenomenological/embodied perspective. Kierans and Maynooth (2001), writing now over 20 years ago, sought to progress thinking from medicalized perspectives of kidney disease towards an embodied perspective, bringing bodies, medicine and culture together, stating ‘it is the sensory body, our ‘existential vehicle’ of experience, which takes precedence over the abstract bodies of medical science or even the theoretic, textual bodies of postmodernism’. Such an approach demonstrates the ongoing ontological tension between biomedical and social/cultural constructions of the body/being in the world.

While Kierans and Maynooth (2001) position the experience of end-stage kidney disease as embodied, here we progress this position by viewing haemodialysis through a posthuman lens. To do this, we use Fox and Alldred’s (2022) ethological tool kit to analyse findings from qualitative research, conducted as part of the NightLife study, focusing on the experience and impact of haemodialysis. This study was planned and conducted from a largely social constructionist perspective. Our secondary analysis of the qualitative findings for this paper, however, follows an ethological approach in much the same way as Cluley et al. (2023a) use to analyse social constructionist research addressing frailty. In doing so we argue that haemodialysis is more than human, it is a phenomenon that involves the relational presence of both human and non-human factors that change over time and have the capacity to affect and create. Important to this assertion is that our approach prioritizes neither human nor non-human factors but rather situates both as intertwined and agential.

Thinking about haemodialysis from a post-human perspective repositions haemodialysis as neither a medical technology that transforms and shapes the bodies of patients undergoing treatment, nor as a socially/culturally constructed event or even as an event experienced solely through the body. Rather, thinking about haemodialysis from a posthuman perspective repositions the research focus away from human agency towards the relationships between human and nonhuman factors. In this way haemodialysis is positioned as a fluid and relational composition or assemblage of human and nonhuman factors that function to create the event that is haemodialysis. Important to this is the capacity for these assembled factors to create both things and experiences that will be both similar and different and can change over time. Understanding haemodialysis as a post-human event presents a holistic platform from which improvements to treatment and care can be made. In offering this approach to understanding both the treatment and the impact of the treatment of end-stage kidney disease, we suggest that such an approach be applied to other treatments and chronic illnesses.

To provide some theoretical context to these assertions we first outline the foundations of posthumanism. We then detail our ethological approach and follow this with a discussion of our findings.

## Posthumanism

A growing number of theorists are associated with posthumanism (Harraway, Barrad, Braidotti, Delanda, Latour, Martin, Fox and Alldred, Bennet). While differing and sometimes contradictory thought processes feature across this work (DeLanda (2016) suggests a realist approach while Barad (2007) suggests a diffractive approach) in the main, post human approaches are premised on a number of shared and overlapping, philosophical foundations. We outline these as five unifying principles. To illustrate these, we draw on literature from key posthuman scholars, we recognize that within posthumanism differences in approach exist, however, here we seek to outline generally shared principles to provide an overview of the posthuman approach to understanding the world and our being in the world.

First, post humanism is post-anthropocentric (Fox and Alldred, 2021). Both human and non-human factors are afforded agential status and capacity for influence and change. The world is neither pre-social nor socially constructed. Rather, being in the world is produced by related and changeable human and nonhuman factors that include a fluid range of elements such as - bodies (human and animal), objects, values, social norms, feelings and memories (Cluley, 2020). The practical outplay of this is seen in Latour’s Actor Network Theory, Deleuze and Guattari’s concept of (or logic of) assemblage, Delanda’s assemblage theory, Barrad’s diffraction and Braidotti’s posthumanism.

Second, posthumanism is premised on what is often described as a flat ontology but is perhaps better described as a bumpy (Cluley, 2020) or zigzag ontology (Deleuze and Guattari, 1987). The flatness stems from the posthuman rejection of essence in favour of multiplicity, whereby no one entity is afforded preference. Rather all things are afforded equal, and importantly, relational being in the world: including, bodies (human and animal), objects, events/experiences/imaginaries, technology, values, social norms and culture. Dualisms, such as mind/body and human/nonhuman, are transcended. Instead of opposition there is relational fluidity, instead of essence there is multiplicity.

Deleuze and Guattari (1987) refer to trees and rhizomes to illustrate these distinctions. Trees grow upwards and have points and positions that make up their structure. Tree like thinking organizes the world according to binary dualisms. Thus, trees are examples of the structural, hierarchical thinking that tends to dominate Western philosophy (Cluley, 2020). Rhizomes on the other hand are reflective of a posthuman approach. They consist of connected nodes, start in the middle without a beginning or an end, and span in differing directions. They connect to other things, other rhizomes, and when broken or fractured rhizomes can reconnect and progress even when challenged (Cluley, 2020).

A posthuman ontology, moreover, is not static. It is fluid and changeable. Importantly, the capacity for change within ‘things’ and the other ‘things’ they are related to is acknowledged. A posthuman ontology involves lines of flight, creation and disruption. To reflect this capacity and motion, a posthuman ontology, as outlined earlier, is better described as a bumpy or zigzag ontology rather than flat. To describe an ontology as flat removes boundaries but also infers sameness rather than change.

Third, posthuman approaches position things/being in the world as relational. ‘Things are never things on their own’ (Cluley, 2020) nor do things remain the same. Relational elements (human and nonhuman) are connected, networked or assembled and in turn connect and disconnect to other networks/assemblages so that all things are in a continual state of becoming (Deleuze and Guattari, 1987). In this way it is acknowledged that nothing is final, rather all things have the potential for change.

Deleuze and Guattari (1987) introduced the concept of assemblage based on their reading of Spinoza, Hume, and Bergson to capture both the complexity and fluidity of things. To return to their example of the rhizome, assemblages function rhizomaticaly with the purpose of creation and change. All elements involved in the creation of something connect fluidly without beginning or teleological end. The focus is the drawing together of elements, the thing that is then produced and the change that results (Cluley et al., 2023b). For Deleuze and Guattari (1987), the assemblage of human and nonhuman elements is brought together through the flow and productive force of desire. Assemblages do not have teleological destinations, rather assemblages and the things they produce move fluidly, contain lines of flight; they have the potential to disable and negate as well as enable and create (Duff, 2014).

Fourth, viewing the world in these terms necessarily means that posthuman approaches reject the notion of the static or final, rather things are thought to be in a constant state of related becoming, imbued with desire and the potential for change (Cluley and Radnor, 2020). In this way, ‘continuously *emergent* via a series of interactive and productive events/assemblages, rather than founded upon stable structures or systems’ (Fox and Alldred, 2022: 625). It is also important to note that becoming is neither specifically human nor nonhuman; it is post human, a creative coming together of both human and nonhuman factors.

Posthumanism does not focus on what a thing is, but rather on what things can do, what connects things, how are things part of other things and how things change (remembering that ‘things’ can be human and nonhuman). Deleuze and Guattari (1987, p. 257) state: ‘we know nothing about a body until we know what it can do, in other words, what its affects are, how they can or cannot enter into composition with other affects, with the effects of another body, either to destroy that body or to be destroyed by it, either to exchange actions and passions with it or to join with it in composing a more powerful body’. It is important to note here, that Deleuze and Guattari use the term ‘body’ to refer to all things not simply ‘corporeal bodies’.

Fifth, asking what a body/assemblage/thing can do, is fundamentally related to the posthuman assertion that assemblages have the capacity to affect. In posthuman terms, ‘affect’ is a change in something. It is the flow or coming together of things in the form of an assemblage that determines what a thing can do/what its capacity is (Fox and Alldred, 2022). Indeed, potential to create and change is intrinsic to assemblages (Cluley et al., 2021). Importantly, this affective potential is a characteristic of both human and nonhuman matter. Affects bring things together, territorializing and deterritorializing. These terms introduced by Deleuze and Guattari (1987) refer to the potential for affective flows to create specification and disruption or as Deleuze and Guattari also state, molar (majoritarian/widely accepted) and minor (minoritarian/other) identities. In a territorialized or molar state, the thing that is assembled gains what has been described as generalized status (Fox and Alldred, 2022). A good example of this would be the medical model of health in clinical settings. Deterritorialization, however, refers to minoritarian status that is typically subject to change but also has the potential to become majoritarian. A good example of this would be the social model of health in clinical settings. As Fox and Alldred state, ‘these two antagonistic movements of specification and generalization mean that the possibilities and limits upon what a body can do (capacities) also fluctuate continuously and unendingly’.

Overall, posthumanism is an approach that surpasses traditional understandings of the world, that accounts for both human and nonhuman interaction and affords both not just equal status but also agency or capacity to affect. Posthumanism acknowledges complexity, fluidity and potential/desire within all things. The analysis presented in this paper is influenced by the posthumanism found in the work of Deleuze and Guattari (1987) and was conducted following Fox and Alldred’s ethological toolkit.

## The ethological toolkit

The use of posthuman theory to conduct empirical research and make sense of empirical research findings is increasing. However, methodological frameworks for doing this are few and far between. As outlined, Delanda proposes a realist application of posthumanism, Barad and Braidotii draw on feminism and Latour’s actor network theory can also be positioned a posthuman (Fox and Alldred book). None of these applications, however, provide a specific methodological framework for conducting empirical research. Fox and Alldred’s ethological toolkit is premised on Delueze’s ethology and provides a means for actioning post-humanism as a methodological approach for research practice. Key concepts include those addressed above – relationality, assemblage, affect, capacity, territorialization and deterritorialization. Fox and Alldred (2022) describe ethology as the study of ‘capacities for affecting and being affected’, and of how these capacities diminish or strengthen a body’s or a thing’s power to act’.

Viewed through an ethological lens, each research project includes an assemblage of research choices relating to subject matter, participants, methodological approach, data collection techniques, use of supporting technologies and theories/concepts (Fox and Alldred, 2022). Fox and Alldred (2022) break the research assemblage down into smaller research machines associated with particular steps in the research process such as generating a research question/hypothesis, ethics, data collection, analysis and dissemination of findings. In this way, as Fox and Alldred (2022: 629) state, each machine enables ‘particular research capacities’ within a particular methodological approach. This point is important to the research findings developed. The particular approach taken and methodological machines at work will influence the findings produced.

An ethological approach to research, moreover, acknowledges the creative capacity of research. For this reason, in order to be operationalized effectively in the research process, Fox and Alldred (2022) assert that an ethological approach should flow throughout the assemblage of a research project, including the generation of research questions, data collection, analysis and writing up. Next we outline how an ethological approach was applied to conduct a secondary analysis of qualitative findings from the NightLife study.

## Methods

The work presented and discussed is taken from an integrated process evaluation of an ongoing clinical study, The NightLife Study (https://www.isrctn.com/ISRCTN87042063). NightLife is a National Institute for Health and Care Research (NIHR) funded, UK-wide, multicentre randomized controlled trial to evaluate the impact on quality of life and clinical effectiveness of 6-months extended-hours in-centre nocturnal haemodialysis (INHD) compared with in-centre daytime haemodialysis (ICHD). The study is composed of five workstreams addressing clinical, economic and experiential factors. The integrated process evaluation explores factors important to the delivery and implementation of the intervention (INHD) using ethnographic methods and photovoice. The current paper draws upon data generated through the first phase including 120 hours of onsite observations, and interviews and photovoice with 35 patients, to build a holistic understanding of the delivery, receipt and impact of ICHD, to be used as comparison with INHD experience.

Observations were carried out by VC at four renal units in England and VC made extensive real-time field notes. These field notes are not included in the analysis presented here but have been drawn upon to set the scene. Participants were recruited opportunistically from these same sites by VC (a Sociologist working on the NightLife study who did not know any of the patients). Eligibility criteria included adults receiving daytime haemodialysis with capacity to provide written informed consent. The study received a favourable ethical opinion from West Midlands - Edgbaston Research Ethics Committee (REC reference: 20/WM/0275) and full Confidentiality Advisory Group (CAG) support (CAG reference: 20/CAG/0136) in December 2020 to support the observational element of the study. Participant information sheets were shared with all participants and written consent was provided by all.

All participants were offered the option of taking part in a semi-structured interview or photovoice and their involvement in each of these methods was explained by VC. The majority of people in receipt of haemodialysis are aged 65 and older (UKRR, 2022). For this reason, we were aware that the photograph collection and sharing methods we had chosen (personal smartphones and instant messaging app) would exclude some participants that is, those who do not own smartphones and/or those who do own them but are not confident using them (see forthcoming paper for more information about this decision and its impact). The combination of observations, semi-structured interviews and photovoice was chosen for two reasons. First, to build a holistic understanding of daytime haemodialysis that captured both human and non-human factors and was as inclusive as possible. Second, to allow choice and maximize participant control. Photovoice originated a participatory action research method to facilitate bottom up research and action social change (Wang and Burris, 1997). Photovoice has since evolved into a flexible visual research method (Cluley, 2017; Cluley et al., 2021). A typical photovoice study involves participants taking photographs of a negotiated subject, sharing the photographs with the researcher and using them to guide a conversation (Wang and Burris, 1997). Importantly, the images are not supplements to a semi-structured interview, rather they are a means to elicitation and deeper understanding (Cluley et al., 2021). The content of the photographs and the participant elicitation that accompanies them are of equal importance. Here photovoice was used to capture the experience and impact of haemodialysis both within and beyond the renal unit. The combination of semi-structured interviews and photovoice in this study has generated a holistic appreciation of daytime, ICHD that captured its complexity.

Interviews and photovoice conversations were carried out at the participants’ bedsides while they were receiving haemodialysis. This is a common approach in haemodialysis research owing to the fact that so much of a participant’s time is taken receiving dialysis and attending additional health related appointments (Kaushal et al., 2022). A flexible topic guide was used for the interviews and photovoice work. For the photovoice work, questions were based on the SHOWeD framework (see Box 1). All interviews and talk about photographs were audio recorded using an encrypted dictation device and transcribed. Transcript data was pseudonymized and the audio recording deleted after 4 weeks.

**Box 1.** SHOWeD question framework.

What do you **S**ee here? What is really **H**appening here? How does this relate to **O**ur lives? **W**hy does this condition exist? What can we **D**o about it? **Additional questions included**: how do you feel about this? What does this mean to you? Was there anything else you would have like to show?

An ethological approach was applied to this work. As outlined above, Fox and Alldred (2022) encourage an ethological approach to be followed from beginning to end of the study.

To ask ethological questions of the research findings generated from the observations, interviews and photovoice, we followed Fox and Alldred’s (2022) ethological toolkit as closely as possible. For analysis of findings Fox and Alldred (2022) outline a specific focus and encourage the use of this for coding. Their proposed coding framework mirrors the central concepts of the toolkit including highlighting human and nonhuman factors; affects on human and nonhuman elements, and capacities including the capacity to aggregate, disrupt, affirm, reduce/constrain and to progress. This framework can then be used as a structure for sharing and reflecting on findings. Fox and Alldred (2022) suggest ethological findings should summarize the human and nonhuman factors involved, highlight the assemblages of relations between the human and nonhuman factors involved focusing on affects and capacities, and provide case studies to illustrate these interactions and their affective capacities. For the findings presented here, we used the proposed ethological coding framework to organize our photovoice and interview data. We used NVivo12 (qualitative analysis software) to support this. VC completed the analysis working iteratively between the transcripts first to identify all references to human factors, non-human factors. These were then listed (a concise presentation of this list is included in the findings section) and the transcripts were then re-coded to identify relationships/assemblages of human and nonhuman factors and the consequent capacities for enablement and restriction. Our findings and discussion are presented below.

When writing up an ethological analysis, Fox and Alldred (2022) propose that findings are presented and discussed to include: (1) a summary of human and non-human factors identified, (2) textual or graphical documentation of the assemblage and (3) the use of case studies to illustrate affective interactions and the capacity of assemblages to produce/change.

## Findings and discussion

In line with Fox and Alldred’s ethological toolkit our findings are organized as follows – first we present the human and nonhuman factors identified across the data. We then illustrate the relational nature of these factors and present haemodialysis in terms of a fluid and relational assemblage that includes lines of flight and affective capacity. Finally, we discuss these affective capacities using examples taken from our data and discuss this in relation to previous literature and the micropolitics of haemodialysis.

### Human and non-human relations

Human relations identified across the data included – patients (bodies, blood, flesh, skin, tears, arteries and veins), taxi drivers, doctors, nurses, dietitians, ambulance drivers, housekeeping staff, friends and family members, transport co-ordinators, technicians and ward clerks.

Nonhuman factors identified included – renal units, haemodialysis machines and the supporting paraphernalia (the artificial kidney, tubing, tape, bandages and dialysate, power points and cables, water treatment and waste disposal), hospital beds and chairs, nurses’ stations, televisions, smartphones, tablets headphones, books, homes, carparks, alarms, pets, food and drink, medication, transport, outdoor spaces, medical notes, workplaces, clocks, sounds and smells.

These factors were both similar and different across participant talk but crucially functioned together as an assemblage to produce the event that is haemodialysis and the affect it has on both human and nonhuman matter. Indeed, it is the affective interactions between human and non-human factors that draw the elements together and instil their assemblage with creative potential (Cluley et al., 2023b). The examples below show elements of this affective relationship.

### Assemblage and affective interaction

Here we illustrate key elements of the haemodialysis assemblage, focusing on the human and nonhuman factors involved and their affective interaction. The following examples are discussed, the dialysis environment, the fistula, and the dialysis machine.

#### The dialysis environment

Participants talked a lot about the dialysis environment and many of the photovoice participants shared photographs within dialysis units to show this. Photographs included images of the dialysis machine, staff, dialysis peers, windows and lighting, beds, nurses stations, the ceiling, corridors, and overbed tables. The dialysis environment, moreover, involved relational, interaction between human and non-human factors with affective capacity. An example of this can be seen in the example taken from Katherine’s interview.

Katherine is in her 60s and has been receiving daytime in-centre, haemodialysis for the last few years. She generally dialyses in the same bed in the same bay surrounded by the same patient peers. Katherine talked a lot about of the environment of the renal unit and found this had a direct effect on her. Katherine talked about the renal unit community and the friendships she had made with patients and nurses alike, she talked about how she spends the 12 hours a week she is on dialysis and the things she brings with her to help pass the time – magazines and colouring books. Katherine also talked about the sensory environment and how this improved and then limited her experience.

‘I didn’t like that they put, oh, stuff on the glass, so you can’t see through the windows. It’s nice to sometimes, it’s only the car park, but it’s quite nice to be able to see outside. And it wasn’t, it wasn’t our doing in here, it was somebody over there, the nurses over there, the District nurses. They put that on. They said that it would be more private if they did that. But I. . . You know, nobody asked us’.

The material change to the windows actioned by others affected Katherine’s experience. Not only did she not feel included in the decision but the ‘stuff on the glass’ changed her visual environment when dialysing. Katherine went on to talk about how this change made her feel more confined to the unit, in that her dialysis environment no longer had the option to look outward, only inward. For Katherine the dialysis environment included both human and non-human elements brought together by their affective interaction. For Katherine, the dialysis environment included herself at the centre of relational factors such as windows, dialysis staff, the hospital bed, the dialysis machine, and other patients. If positioned as an assemblage of human and non-human factors, moreover, the dialysis environment can be seen to have a palpable and changeable affect on patients.

**Figure 1.**
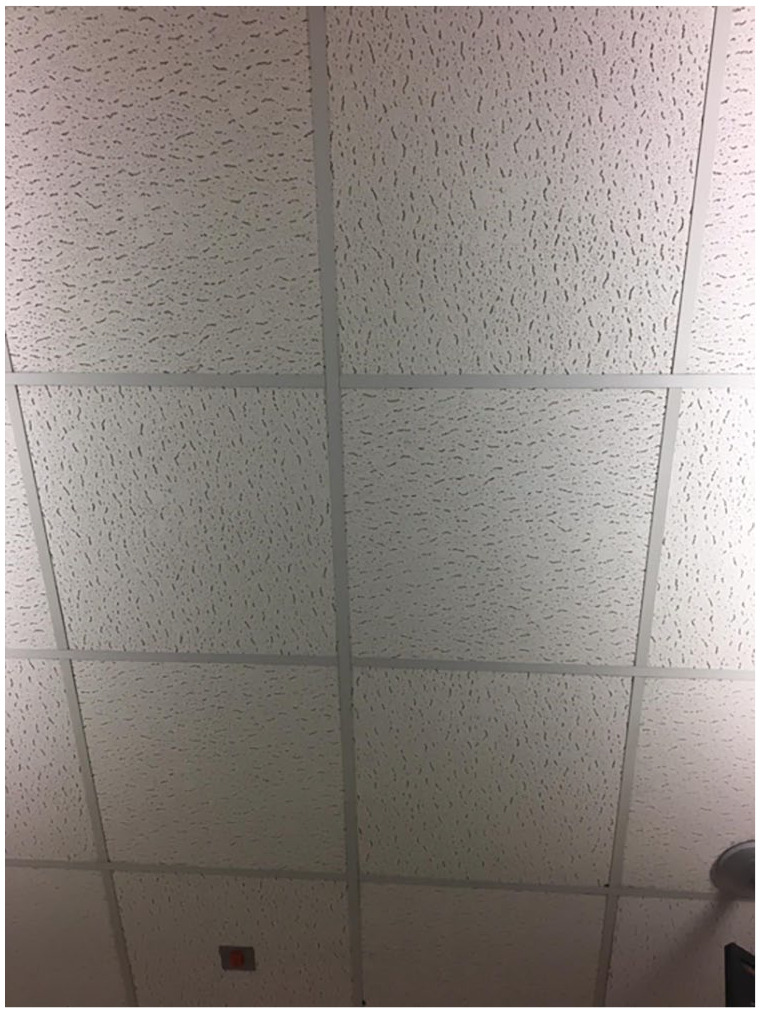
Marisol’s view.

Another example of the palpable affect of the dialysis environment can be seen in Marisol’s photovoice images and talk. Marisol talked about a range of factors that assembled to create her dialysis environment. These included smells, the ceiling, poor wifi, televisions, noise, the bed and exercise equipment. For Marisol, the dialysis environment was essentially boring. Marisol shared a photograph of the ceiling above the bed that she dialyses in to show her usual view.

When talking about the sensory environment of the dialysis unit Marisol said

‘Its really loud here. The machines alarm a lot. He’s [patient in the neighbouring bed] got the TV on really loud. So I know everything about Coronation Street. I never say anything to him, because its like his space as much as my space. Last week it was horrible, a patient shat themselves and the smell, it was really not nice, they put the curtains around, but. . . .it was just horrible. So something like that, its horrible, whereas listening to a bit of Coronation Street, I really don’t care’.

In this example various human and non-human factors a drawn together through their affective interaction to create the dialysis environment that Marisol describes. These factors include staff, the dialysis machine, sounds, smells, patients, ceilings and curtains. The interactions between these elements and their fluid relationship has an affective impact on Marisol who, through her talk, is positioned at their centre. Marisol’s example highlights the fluid nature of assemblage. One week a smell affected Marisol’s experience of dialysis, while the week before, the smell did not feature.

**Figure 2.**
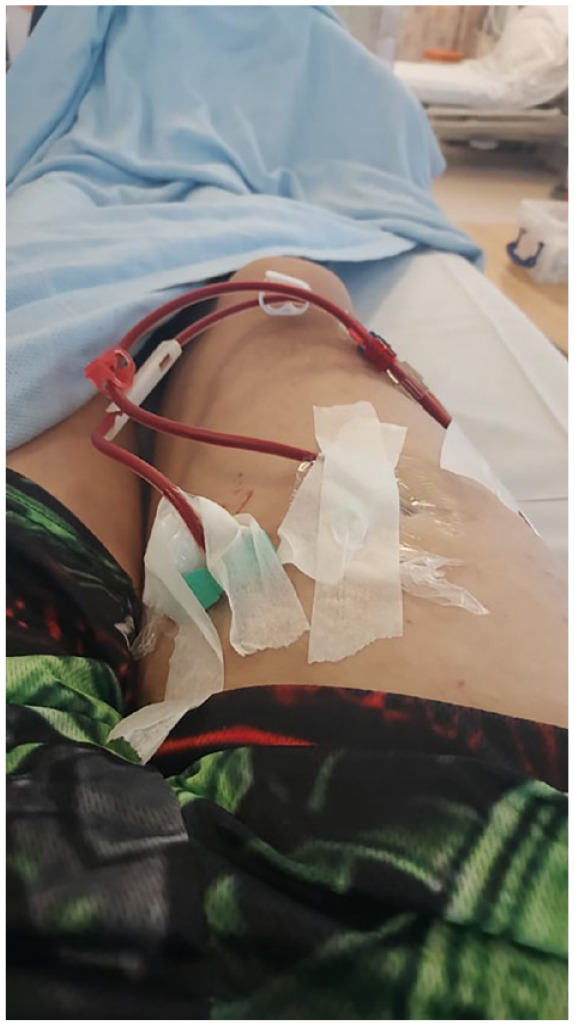
Nick’s graft.

#### The fistula

For dialysis to occur the patient’s blood needs to be effectively and efficiently accessed and diverted through what is known as the dialysis circuit. This is typically done either through the surgical creation of a fistula (the joining together of a vein and an artery) a graft, or a tunnelled catheter. The dialysis circuit involves the removal of blood from the body (using needle insertion for an arteriovenous fistula or graft, or direct connection of lines for tunnelled catheters), blood flows through the specialized plastic tubing/lines, through the dialyser (artificial kidney), with dialysate flowing in the opposite direction, ‘clean’ blood is returned to the body and the waste products are drained away.

Typically, this connection of bodies, needles, tubes and machine is carried out by a dialysis nurse, however, some patients choose to receive training to do it all or in part themselves. Participants discussed this process in-depth. For those who had been on dialysis a long time, some had been in receipt of dialysis upwards on 20 years, numerous fistulas had been required. With a failed fistula and the creation of new fistulas comes the production of scar tissue. This can have a visual effect on the patient’s body, leaving them with large, raised, lumps that protrude up through the skin. Nick, a photovoice participant in his 40s, took a photographs of the graft he has in his upper thigh.

Nick said,

Well, well I did have one [fistula] in me arm. A while ago. But what happened, big lumps on my arm, so what they did, they had to remove them. Then they tried to build a graft as well. And it didn’t take. And my belly, like my stomach like is like a motorway at the minute I’ve lost count of how many operations I have had! [Laughs].

Participants talked about how their fistulas affected their relationship with their bodies and how they used them.

Katherine said,

One of the things I’m not very keen on is I’ve got what they call a fistula. Where they put two veins together. They say you ought to put your fingers on it ever so often, just to listen, I think they call it a ‘thrum’ or something. And I don’t do that because I don’t like the feeling. But sometimes, you know when you put your arm up, like that, you can hear it, you know. Oh yeah, and that, oh no! A bit like, it’s a bit like, running fingers down a blackboard. Not nice, I don’t like that. And, also I think what I don’t like is the fact that it looks like I’ve got a slug, at the moment, underneath the skin. Because obviously it’s, it’s, it’s raised. And I see some people’s and they’re awful, they’re really bulging. You know, the vein and what have you. That I don’t like.

In the micro assemblage of the fistula, moreover, human and non-human factors, such as veins, blood, tubes, needles, the dialysis machine and nurses, are drawn together through affective interactions with the patient’s body both during haemodialysis and in everyday life.

#### The dialysis machine

For effective dialysis, on average, patients are connected to a dialysis machine for 3–5 hours a time, 3–4 days a week. Haemodialysis is a systematic procedure that requires knowledge of both the machinery and of a range of physiological and biological factors for success such as patient weight, potassium and sodium levels, and calculations of how much fluid to remove. Many of the participants were very knowledgeable about their treatment, some could connect themselves to the machine, some could adjust the dialysis machine settings, others knew how to deal with alarms raised by the machine. In contrast some participants had limited understanding of their treatment and personal health information. Regardless of level of knowledge and understanding, the participants talked a lot about their dialysis schedule, the dialysis machine and their bodies in relation to the machine.

Amanee who had been receiving dialysis for nearly 30 years talked about changes to the dialysis machine over this time and the affect this had on her body. Amanee was able to connect herself to the machine and was able to programme it to remove the correct amount of fluid. While talking to VC various alarms sounded from the machine and Amanee was able to respond to these. In contrast, Silas who had been receiving dialysis for 18 months said

‘They weigh you before your come in. Say ‘well how much shall we take off’ to get your what do they call it ‘ideal weight’ so when they connect you to the machine they can program in the information’.

Similarly, Aadhan said

‘I don’t read the screen [on the dialysis machine]. I don’t know what’s involved but the nurses come round and they sort it out if the alarm goes off’.

Participants described the dialysis machine as ‘a life saver’ (Dorothea), ‘part of them’ (Joyce), ‘very clever’ (Margaret), ‘a ball and chain’ (Heather), ‘a surreal sight’ (Benjamin) and ‘a male because its always grumbling’ (Amanee). Being connected to a machine, moreover, extended beyond the relationship between body and machine and included additional human and non-human factors such as nurses, screens, sounds, scales and measurements.

In the examples above, it can be seen that a range of human and non-human factors that are both similar and different depending on individual circumstances, assemble together to create the event and experience that is incentre, daytime, haemodialysis (ICDHD). It is important to note that none of the assemblages that make up the haemodialysis assemblage are static. Rather they are fluid and will involve different factors at different times depending on circumstance. The research drawn on here, for example, was conducted during the COVID-19 pandemic. Prior to this, clinical staff and patients did not wear masks and the beds were located closer together. This allowed the participants to talk freely with staff and other patients and many spoke about the relationships they had built over time. The addition of masks and the relocation of beds affected the development and maintenance of these relationships.

### Affective capacities

Specific elements of the dialysis assemblage had differing affective capacities. Affective capacities can have social, bodily, environmental, psychological and cultural affects that can both enable and constrain (Cluley et al., 2023b). Drawing on the sample of the fistula, seen above, having a fistula had both bodily and social affects. For many, having a fistula elevated their sense of caution and the how they felt they now could or couldn’t use their bodies. Junior and Matis had given up work, Katherine gave up playing tennis, golf and badminton, Theodore gave up lifting weights, Sam’s parents now felt he shouldn’t carry heavy shopping bags, Hema couldn’t sleep because of it, and Dorothea had given up driving and said

I have lost my daily routine, shopping and going out on my own. I can’t do that now, because I gave up driving [because of her fistula], so everywhere my husband has to come. Like yesterday, Saturday, there was a get together for a barbecue. But it was so far, I couldn’t go. Before I would have gone by myself.

The construction of a fistula, to aid the efficacy of dialysis had the capacity to both facilitate and restrict life. Emotions such as enjoyment, independence and pleasure were constrained by the affective capacity of the fistula to create a sense of fear within both the patients and their friends, family and employers.

Similarly, as seen in the dialysis machine and dialysis environment examples, participants talked a lot about the dialysis machine and its capacity to give life while restricting the lived experience of life and confining it to limited spaces – mainly the dialysis unit and their own homes. Following a dialysis session, many participants were left feeling tired and when back at home reported spending the remainder of the day resting. The majority of the photovoice participants’ photographs mirrored this spatial limitation in with the vast majority of photographs being taken in the home. These photographs tended to show bedrooms, living rooms, sofas, chairs and televisions.

When talking about their time after dialysis participants said:

I come back home, take my medication, my blood pressure goes down. I feel extremely tired, so I take like a nap, watch some TV. Actually on dialysis day I stay indoors for all day. Sometimes I play games on the PC (Matis)

the thing is that sometimes it may be fine while I’m dialysing here, and then I go home in the evening and my whole evening is ruined because my chest is in pain, pain in my tummy, my head. .’ (Odette)

Indeed, the demands of dialysis (including temporal, bodily and material demands) collated to cause a number of the participants to move in with parents or siblings following previous independence. The participants talked about this paradoxically, both acknowledging the support this provided but lamenting their loss of independence (Cluley et al., 2023a).

The pervasiveness of haemodialysis and its side effects moreover, had a range of affects that infiltrated into participants’ lives outside of the renal unit. The examples provided illustrate the affective capacity of the assemblage that is haemodialysis. It is important to note that this capacity is fluid and is likely to differ for individual patients depending on other assemblages related to context and circumstance. In the dialysis environment example, for instance, Katherine’s experience of haemodialysis was changed by the addition of frosting on the windows. In other dialysis units attended by VC, there were no windows and in another an entire wall was transparent glass. In this particular unit, a participant likened their experience to being in an airport lounge and others complained that on sunny days the unit was too bright and too hot. Importantly, the assemblage of haemodialysis (the related individual human and non-human factors drawn together by affective interaction with the capacity to create), is fluid and will have a different composition for all patients resulting in differing affects. Likely there will be similarities but there will also be differences. Additionally, the assemblage will change over time depending on circumstance. For Deleuze and Guattari (1987), assemblages do not exist on their own, rather they are related to or plugged in and out of other assemblages that also have affective capacity. As assemblages change over time so too will their affects. This recognition of difference is not a weakness, rather it is a celebration. It is not the case that in recognizing everything, nothing actually matters, rather a post-human starting point highlights the relationships between human and nonhuman matter and the potential for change and affect that such assemblages create. Assemblages are not neat, static packages that can be identified as universal to all experience, they are heterogenous with no beginning or end. Just as rhizomes connect, disconnect and reconnect with other rhizomes, so too do assemblages and the micro assemblages they are part of. In our paper Cluley et al. (2023a) we detailed the disruptive impact haemodialysis can have on lives lived outside of the renal unit. In the examples shown here, haemodialysis and its disruptive affect, moreover, is revealed as a fluid and heterogenous assemblage with the capacity to both enable and constrain, providing life while restricting how it can be lived.

## The significance of an ethological approach

As outlined, the purpose of an ethological approach to research is to open research to a posthuman agenda whereby fluidity and complexity are emphasized over essence and structure, and both human and nonhuman factors are afforded relationality and agency.

In applying an ethological approach to the photovoice and interview transcripts from the NightLife study, haemodialysis can be seen to be more than a relatively standardized and disruptive clinical process. It is also revealed as more than human and more than experiential. While the participants place themselves at the centre of their talk, their photographs and talk reveal more than this. The examples provided show haemodialysis to be a heterogeneous event that involves the assemblage of human and nonhuman factors and has affective capacity that extends beyond the renal unit, beyond bodies and into the complex lives of patients and the environments and relationships they occupy.

The majority of previous studies exploring the experience of haemodialysis have focused on bodily affects such as symptom burden, self-management and patient activation. As outlined some studies have addressed the wider impact of haemodialysis such as unemployment and reduced social lives (Roberti et al., 2018) and some have approached dialysis from a phenomenological, embodied approach, however, these studies maintain and anthropological focus (Kierans, 2005; Kierans and Maynooth, 2001). In adopting a post-human approach the complexity of haemodialysis and all of the factors involved in its affective capacity can be accounted for. This allows for the opening up of the understanding of haemodialysis as an event and an experience, to allow it to be seen as fluid and changeable rather than a static process. A posthuman, ethological approach also allows haemodialysis to be repositioned from something that is done to bodies to something that affects individually and heterogeneously and will likely change over time.

## Conclusion

Overall, the application of an ethological approach to our interview and photovoice findings reveals the complexity, fluidity and affective capacity of haemodialysis in a way that cannot be achieved using traditional approaches to research. The assemblage of human and nonhuman factors illustrated as involved in the event and experience that is haemodialysis can be seen to have had a range of lived affects dependent upon other assemblages unique to individual circumstances. For example, where some participants had returned to living with parents and siblings others, with some adaptations to home life, had been able to remain living independently. While affect was different for different people, our findings highlight one shared affect – enablement and constraint. The arrangement and contents of the assembled human and non-human factors were different depending on individual circumstance, however, all participants experienced a paradox where by life was given by haemodialysis yet constrained by its demands and side-effects. We reached this conclusion in Cluley et al. (2023a). In taking a post-human approach we add to this finding by removing the anthropocentric focus and allowing complexity and agency (including objects) to be foregrounded.

Importantly, the ethological approach used has allowed us to reposition haemodialysis from something that is done to bodies or something that is experienced by bodies, towards an event that affects individually and heterogeneously, that is complex fluid and changeable and involves the assemblage of human and nonhuman factors. This repositioning essentially allows further understanding of the affects of chronic illnesses and their supporting treatments that further allows for a holistic appreciation upon which improvements to delivery and receipt of care can be based. We recommend that research to understand the impact of other chronic illnesses and their affects would benefit from an ethological approach.
